# Myths and facts about alcohol use disorder: a Delphi consensus study

**DOI:** 10.1093/braincomms/fcaf035

**Published:** 2025-01-27

**Authors:** Sophie Hytner, Daphne Josselin, David Belin, Owen Bowden Jones

**Affiliations:** Department of Psychology, University of Cambridge, CB2 3EB, Cambridge, UK; Department of Psychology, City, University of London, London EC1 V 0HB, UK; Department of Psychology, University of Cambridge, CB2 3EB, Cambridge, UK; Department of Psychology, University of Cambridge, CB2 3EB, Cambridge, UK

**Keywords:** public stigma, alcohol use disorder, Delphi method, expert consensus, anti-stigma interventions

## Abstract

Educational interventions that counter myths about alcohol use disorder with facts have the potential to reduce public stigma. Few such interventions have hitherto been rigorously developed. Using a Delphi expert consensus method, this study identified myths and facts to include in an intervention targeting the public stigma of alcohol use disorder. Sixteen UK-based experts (four academics, five clinicians and seven experts-by-experience) completed three sequential online survey rounds. The first round was used alongside a systematic review of the literature on public alcohol use disorder stereotypes to develop 13 myth-fact pairs, which participants quantitively scored in subsequent rounds to determine their importance for inclusion. Pairs reaching consensus (>70% agreement) on high importance (mean score, 7–9) challenged beliefs that alcohol use disorder ‘*only affects certain groups’*, and that people with alcohol use disorder ‘*cannot recover’*, are ‘*to blame’* for, and ‘*able to control’*, their drinking. The myth-fact pairs scored as most important relate to responsibility- and recovery-based themes and provide a basis for future educational interventions for public alcohol use disorder stigma.

## Teaser

Hytner *et al*. used a Delphi method to establish expert-led myths and facts to challenge alcohol use disorder stigma. Input from both professionals and experts-by-experience and a literature review identified important themes relating to responsibility and recovery that contribute to the perpetuation of alcohol use disorder stigma.

## Graphical Abstract

**graphical abstract:**
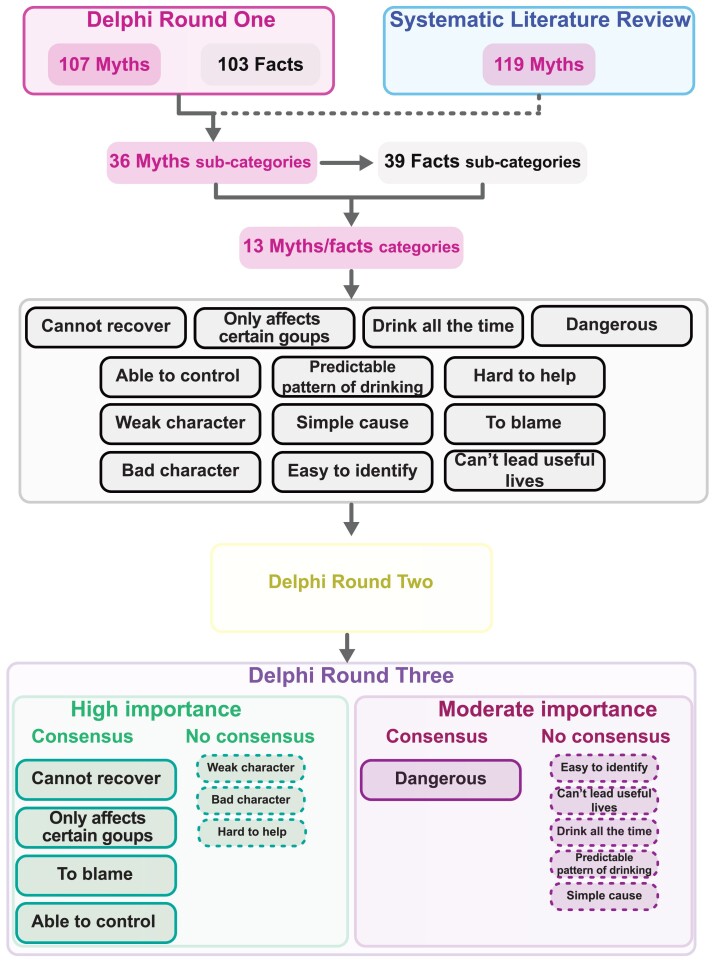

## Introduction

Public stigma is a process of social devaluation whereby people are labelled, linked with negative stereotypes and emotions and discriminated against.^1^ This can lead to profoundly detrimental consequences, especially for individuals with a psychiatric disorder such as a substance or alcohol use disorder. For instance, public stigma has been shown to impair recovery from alcohol use disorder (AUD). Individuals with AUD who perceive public stigma may develop internalized stigma, which can affect treatment outcomes through the worsening of negative emotions and alteration of cognitive mechanisms necessary for the maintenance of abstinence, such as self-efficacy.^2^

Despite its negative impact, the public stigma of AUD remains highly prevalent,^3^ being relatively resistant to anti-stigma campaigns, at least in the UK.^4^ Meanwhile, few evidence-based interventions are available effectively to challenge it. A need has, therefore, been identified to develop public anti-stigma interventions for AUD, drawing on established mental health stigma-reduction strategies.

‘Education’, an anti-stigma strategy commonly used in public mental health campaigns, counters inaccurate stereotypes (‘myths’) about mental illness with facts.^5^ The premise of the ‘Education’ strategy is that challenging negative attributions about people with a stigmatized condition (e.g. ‘weak’) can reduce the public’s negative emotions (e.g. anger) and discriminatory behaviour (e.g. social rejection) towards them.

Education compares favourably with other key stigma-reduction strategies. Its effects have been found to be greater than ‘protest’,^6^ which condemns stigmatizing beliefs, and equal to ‘contact’, which promotes positive interactions between the public and people with neuropsychiatric disorders.^7^ However, evidence for myth-fact interventions addressing public AUD stigma is underdeveloped. While one UK study assessed a factsheet in this context, finding it ineffective,^8^ the intervention’s development process and messaging were unclear, thereby limiting further evaluation.

Intervention development represents a distinct pathway of evidence generation. Accordingly, the present study aimed to clearly outline the development phase of a myth-fact intervention for public AUD stigma in the UK. Given the scarcity of recent population research on UK public stereotypes of AUD,^4^ myths and facts to include in an anti-stigma intervention were gathered using a Delphi approach.^9^ It enabled the generation of myths and facts based on both a synthesis of the literature and the views of a development team with relevant expertise. The study used three rounds of online surveys to identify (i) common public stereotypes (‘myths’) about AUD and corresponding facts and (ii) their perceived relative importance for inclusion in an anti-stigma intervention for public AUD stigma. Its purpose was to generate a prioritized list of myths and facts about AUD for future intervention research.

## Materials and methods

### Ethics approval

The study was approved by City, University of London's Ethics Committee. The procedures used in this study adhere to the tenets of the Declaration of Helsinki.

### Consent to participate

Informed consent was obtained from all individual participants included in the study.

### Design

The study employed a mixed-methods Delphi design, which enables structured group communication across multiple iterative survey rounds to generate consensus among an expert panel. The Delphi method was suitable for identifying myths and facts about AUD, given its utility for addressing gaps in extant literature. A mixed-methods approach allowed the generation of varied myths and facts that could then be scored according to their perceived importance for inclusion in an anti-stigma intervention.

#### Panel recruitment

Three groups of panellists with professional expertise in, or personal experience of, AUD were recruited through purposive and snowball sampling: academics, clinicians and experts by experience. Panellists were required to speak English, live in the UK, be 18+ and have expertise relevant to their group.

(i) Academics: researchers with at least one publication on the topic of AUD
(ii) Clinicians: mental health practitioners registered/accredited with a professional regulatory body (e.g. HCPC, BACP) with at least 1 year’s experience working with AUD
(iii) Experts-by-experience: lived experience of AUD with at least 2 years’ abstinence, a time at which relapse rates have been found to decline.

Social media adverts were placed on an online network of addiction professionals to recruit academics and clinicians. Additionally, emails were sent to academics working in relevant university departments with published work in the field of AUD (identified by titles and abstracts) and clinicians working within third-sector and NHS centres for alcohol treatment (identified by centre managers). Recruitment information for experts-by-experience was distributed at London Alcoholics Anonymous meeting locations.

#### Procedure

City University of London's Ethics Committee approved the study, which was completed online. Eligible participants provided informed consent and demographic information. They then completed, over a 4-month period, three anonymous survey rounds, which is usually enough to reach a consensus.^9^ Round one (∼30 min) gathered qualitative insights on common myths and facts about AUD. These were used alongside a systematic literature review of studies exploring public attitudes towards AUD to form a list of myth-fact pairs. Panellists then quantitatively ranked the pairs in Rounds 2 and 3 (∼10 min) based on their importance for inclusion in a public anti-stigma intervention.

**Figure 1:**
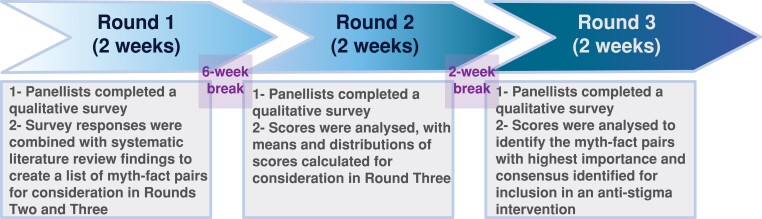
Flowchart of Delphi procedure to establish myths and facts to include in an educational anti-stigma intervention.

Debrief information followed all rounds. Each survey was piloted before data collection and had a 2-week response window with up to three reminders. A 6-week break after Round One and 2-week break after Round 2 facilitated analysis and item refinement. Figure 1 presents the study’s procedure.

### Materials

Panellist materials were hosted on Qualtrics, a secure online survey platform. The study aimed to focus on severe AUD, since stigma tends to be stronger for more severe neuropsychiatric disorders. The term ‘alcohol dependence’ was used in the materials to depict severe alcohol difficulties with accessible language. This was defined as a pattern of alcohol use involving features such as impaired control, salience, negative consequences, tolerance and withdrawal.

#### Demographic

Participants completed demographic information including age, gender, ethnicity and, for academics and clinicians, job role and organization type.

#### Round one survey

The Round One survey (Supplementary Fig. 1) asked panellists to list, based on their expertise/experience: (i) up to 10 common myths about AUD and/or people with the condition and (ii) corresponding facts to challenge each myth.

#### Round 2 survey

The Round 2 survey (Supplementary Fig. 2) was developed based on (i) qualitative analysis of Round One responses, (ii) integration of incremental themes from the systematic review and (iii) additional facts about AUD drawn from the scientific literature. Panellists were invited to score 13 myth-fact pairs using a 9-point Likert scale (ranging from 1 = ‘not at all important to include’ to 9 = ‘very important to include’ in an intervention for public AUD stigma).

#### Round 3 survey

The Round 3 survey (Supplementary Fig. 3) asked panellists to re-score each myth-fact pair from Round 2 using the same scoring method, while considering the mean and distribution of other experts’ Round 2 responses. Experts were informed that they did not need to conform to the average view and could explain reasons for revised scores using optional free text boxes alongside each pair.

### Data and statistical analyses

All data were processed and analysed with Microsoft Excel.

#### Systematic literature review

**Figure 2:**
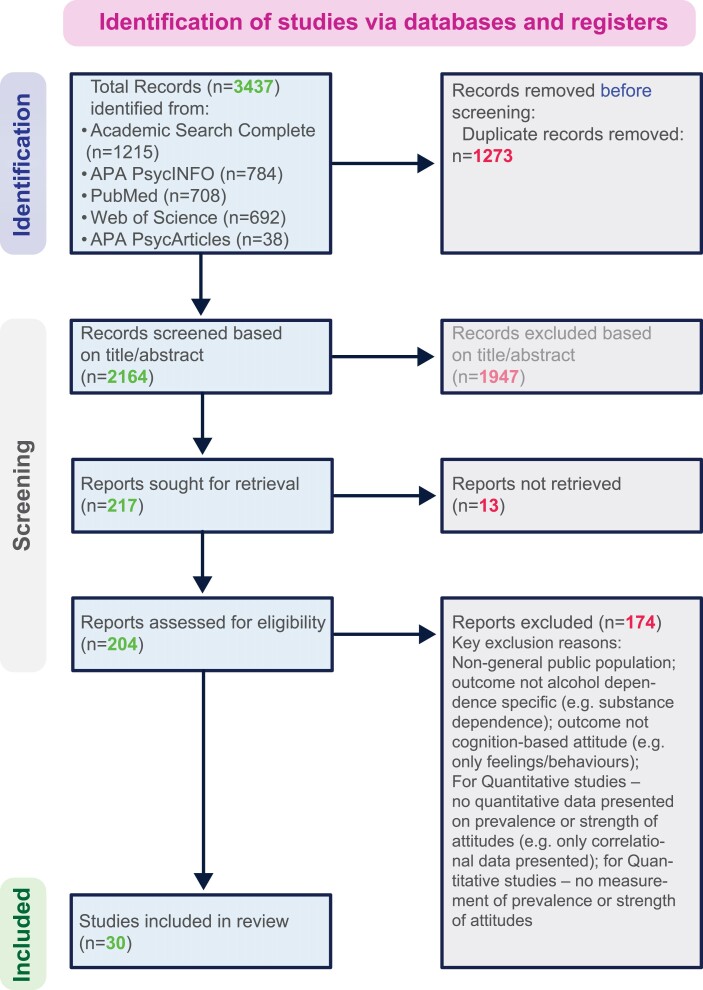
Summary of systematic literature review: process for identification of studies detailing public stereotypes of AUD.

A systematic review of studies on public attitudes towards AUD was conducted to identify commonly endorsed stereotypes. As illustrated in Fig. 2, five electronic databases, namely Pubmed, PsychINFO, PsychArticles, Web of Science and Academic Search Complete, were searched on 11th November 2021, filtered for English language and 2001–2021. The following complex search was used: (addiction* OR alcohol* OR ‘alcohol abuse’ OR ‘alcohol dependen*’ OR ‘alcohol addict*’ OR ‘alcohol use disorder*’ OR substance* OR ‘substance abuse’ OR ‘substance depend*’ OR ‘substance addict*’ OR ‘substance use disorder*’) AND (attitud* OR belief* OR attribute* OR stereotype* OR myth*) AND (stigma*) AND (public OR ‘general public’ OR population*). Retrieved articles were exported, with duplicates removed using RefWorks. Their titles were screened against inclusion and exclusion criteria. Articles were required to be in the English language and to study: (i) Population: adult (16+) members of the general public, or experts-by-experience/healthcare professionals offering perceptions of public stigma; (ii) outcome: public attitudes (e.g. stereotypes, views, beliefs) towards AUD; (iii) design: quantitative measurement of attitude strength or prevalence, using a mean Likert score (e.g. on a disagree-agree scale) or percentage of respondents agreeing with an attitude; or qualitative assessment of attitude existence, nature or prevalence (e.g. using focus groups or interviews).

The full texts of articles meeting eligibility criteria or where initial screening was inconclusive were read. Those that, on further examination, did not meet eligibility criteria (or where important information was not available) were excluded. The literature search yielded 30 articles across 13 countries, presented in Supplementary Table 1.

To compare experts’ views to the existing literature, stereotypes in the review were classified as either ‘endorsed’ or ‘not endorsed’ by the public. They were considered endorsed in qualitative studies if they were held (or perceived to be held) by participants, and in quantitative studies if they had a mean Likert score above the mid-point (agree or above) or above 50% agreement. This methodology was reversed for non-stigmatizing attitudes (e.g. ‘a person who has had alcohol treatment is just as intelligent as the average person’).

#### Round one

Round One data were analysed using inductive qualitative content analysis. This allowed quantification of the most frequently occurring themes in the dataset. The following process was used^10^:

- Preparation: ‘Myths’ and ‘facts’ were selected as ‘units of analysis’. These were read and re-read (‘immersion’) to get a sense of the whole.
- Organizing and abstraction: Initial headings were attributed to each unit to describe its content (‘open coding’). ‘Grouping and categorization’ was then conducted. First, myth headings representing similar concepts were grouped into myth subcategories. Attitude statements from the systematic review were classified into already established myth subcategories or used to generate additional ones where relevant. Next, myth subcategories with similar content were abstracted into higher-order categories. Fact headings within each myth subcategory were then grouped into fact subcategories. Numerous myth and fact subcategories were generated to enable the choice of themes for myth-fact content.
- Reporting: Frequencies of myth and fact subcategories were counted, with those most frequently occurring prioritized in the wording of myth-fact pairs presented in subsequent surveys. To supplement missing fact sources in the data, additional facts supporting relevant fact subcategories were retrieved from online sources (e.g. journal articles from online databases and national statistics publications).

#### Rounds 2 and 3

Quantitative scores were weighted to give equal weight to each expert group’s views. Data were analysed using descriptive analysis of central tendency (mean) and distribution (frequency table with percentage of responses per Likert score). Myth-fact pairs were then categorized based on their mean importance score into low (below 3), moderate (3–7) and high (above 7), and separately into consensus (≥70% within three-point range on the Likert scale) or non-consensus (<70% within three-point range) based on the distribution of responses. Seventy percentage was selected as a consensus threshold in line with other Delphi studies. Round 3 free text responses were brief and thus summarized thematically without formal qualitative analysis.

## Results

### Panellists

Twenty-one panellists meeting eligibility criteria were recruited between November and December 2021. Eighteen panellists completed Round One (5 academics, 5 clinicians and 8 experts by experience), 16 of which (4 academics, 5 clinicians, 7 experts-by-experience) completed Rounds 2 and 3, demonstrating an 89% retention rate. The study finished in March 2022. An overview of the experts’ demographic characteristics is presented in **Table 1**.

**Table 1:** Panellist demographic information

	Round 1	Round 2	Round 3
Panellists (*n*)	18	18	16
Academics	5	5	4
Clinicians	5	5	5
Experts-by-experience	8	8	7
**Age**			
Under 25			
25–34	11%	11%	13%
35–44	44%	44%	38%
45–54	28%	28%	31%
55–64	11%	11%	13%
65+			
Prefer not to answer	6%	6%	6%
**Gender**			
Male	56%	56%	63%
Female	39%	39%	38%
Non-binary male	6%	6%	0%
**Ethnicity**			
White—English/Welsh/Scottish/Northern Irish/British	72%	72%	75%
White—Irish	6%	6%	6%
Other White background	17%	17%	13%
Asian/Asian British—Indian	6%	6%	6%

Numbers may not sum to 100% due to rounding.

### Round one

One hundred nineteen myth statements from the systematic literature review and 107 myth statements from Round One were condensed into 36 subcategories and 13 categories. One hundred three fact statements from Round 1 were summarized in 39 subcategories. Supplementary Table 2 presents the categories and subcategories. Following analysis, myth-fact pairs (**Table 2**) were generated for expert review in Rounds 2 and 3.

**Table 2:** Myth-Fact pairs based on round one analysis

Category	Myths	Facts
**Cannot recover**	People cannot recover from alcohol dependence	People can recover from alcohol dependence and go on to lead fulfilling lives. Over half of people in treatment for alcohol use in alcohol services in England stop or reduce their drinking after 6 months of treatment.
**Only affects certain groups**	Alcohol dependence only affects certain types of people, like the homeless	Anyone can become dependent on alcohol, regardless of their age, gender, ethnicity or background. Most people with alcohol dependence in England are in employment and stable housing.
**Drink all the time**	People with alcohol dependence drink all the time	People with alcohol dependence have different drinking patterns and drink at varying times. People can be dependent on alcohol without drinking daily or in the mornings.
**Able to control**	People with alcohol dependence could stop or control their drinking if they wanted to	Alcohol dependence leads to changes in the brain that can limit a person’s control over their drinking. For example, when a person becomes alcohol dependent, their brain adapts to heavy alcohol use and can start to need alcohol to maintain its chemical balance. If a person with alcohol dependence stops drinking, this balance can be disrupted and the person may experience harmful symptoms, like anxiety, shaking or seizures. This means that while many people with alcohol dependence try very hard (often repeatedly) to stop drinking, it can be very difficult, and in some cases unsafe, for them to do so without support.
**Drink in predictable ways**	People with alcohol dependence drink in predictable ways	People with alcohol dependence drink different types and amounts of alcohol, and drink in various contexts, including alone and socially.
**Hard to help**	People with alcohol dependence are hard to help	Lots of approaches are helpful for people with alcohol dependence and can be beneficial while people are still drinking. Psychological therapy, medical treatment, self-help groups and support from partners, family and friends, can all aid a person’s recovery from alcohol dependence.
**Weak character**	People with alcohol dependence are weak-willed	People with alcohol dependence can demonstrate high levels of self-control and resilience in other areas of their lives—many work in high-pressure jobs, such as doctors or lawyers. Given changes to the brain and body from dependent drinking, people with alcohol dependence use great strength to stop drinking and stay sober.
**Simple cause**	Alcohol dependence has one simple cause, like trauma or genetics	Everyone’s path to developing alcohol dependence is different, and there are multiple factors that can influence the course of alcohol dependence.
**To blame**	People with alcohol dependence are to blame for their problems	A person’s risk of developing alcohol dependence is influenced by lots of biological, psychological and social factors, many of which are outside their control. Biological factors can include a person’s genetics—approximately 50% of the risk for developing alcohol dependence is explained by genes. Psychological factors can include mental health problems (like depression or anxiety) and personality traits (like impulsivity). Social factors can include trauma (like childhood abuse); social isolation; poverty; unemployment; or discrimination. These factors influence each other and can combine to increase someone’s chance of becoming alcohol dependent.
**Bad character**	People with alcohol dependence don’t care about others	Those with alcohol dependence tend to feel guilty and ashamed about the impact of their actions on other people; and caring about others is a key factor that can lead people to seek treatment. A large number of people with alcohol dependence support other people—81% of those attending Alcoholics Anonymous groups in the UK volunteer their time to help others.
**Easy to identify**	You can tell if someone is alcohol dependent	There may be no visible signs that someone is alcohol dependent.
**Cannot lead useful lives**	People with alcohol dependence can’t lead ‘normal’ or ‘useful’ lives	A significant number of people with alcohol dependence are successful and function well in multiple areas of their lives despite their alcohol use.
**Dangerous**	People with alcohol dependence are a danger to others	Many people with alcohol dependence drink away from others; and many are never violent. A large proportion of people with alcohol dependence is or have been victims of violence and drink as a way of coping with these experiences.

### Rounds 2 and 3

#### Round 2

In Round 2, 9 of the 13 myth-fact pairs were given a high importance score, while the remaining four scored were given a moderate importance score. The highest mean score (‘Cannot recover’) was 8.5, and the lowest (‘Drink in predictable ways’) was 5.9. Consensus levels were typically above 50%, ranging from 100% (‘Only affects certain groups’) to 51% (‘Drink in predictable ways’). Consensus was the highest in the nine high importance myth-fact pairs, with all reaching consensus (70% of responses).

#### Round 3

Experts re-scored all myth-fact pairs in Round 3 since none had received a low importance score in Round 2. Of Round 2’s nine high importance myth-fact pairs, four (‘Cannot recover’; ‘Only affects certain groups’; ‘To blame’; ‘Able to control’) retained a high importance score, which overall increased in Round 3. Two (‘Bad character’, ‘Weak character’) remained high but slightly decreased; and three (‘Easy to identify’; ‘Can’t lead useful lives’; and ‘Drink all the time’) decreased to moderate. Of the four pairs with a moderate importance score in Round 2, ‘Hard to help’ moved to the high importance category in Round 3, while three (‘Dangerous’; ‘Simple cause’; ‘Drink in predictable ways’) retained their moderate importance score.

Six of the 10 myth-fact pairs reaching consensus in Round 2 decreased in consensus in Round 3. However, the four highest importance pairs (‘Cannot recover’; ‘Only affects certain groups’; ‘To blame’; ‘Able to control’) increased in consensus (all above 90%). The fifth highest importance pair (‘Bad character’) slightly decreased in consensus from 72% to 62% in Round 3. Even though consensus was lower for moderate importance pairs, it was reached for two (‘Drink all the time’ and ‘Dangerous’).

**Table 3** presents results from Rounds 2 and 3 and a comparison with endorsement scores from the systematic review.

**Table 3:** Key findings from Delphi Rounds 2 and 3 and comparison with systematic literature review

	Round 2		Round 3		Systematic literature review		
Myth-fact pair category	Mean importance score	% consensus	Mean importance score	% consensus	Endorsed	Not endorsed	Net
Cannot recover	8.5	87%	8.7	100%	0	3	−3
Only affects certain groups	8.5	100%	8.6	100%	4	4	+0
To blame	8.3	93%	8.3	100%	17	5	+12
Able to control	7.9	83%	8.0	92%	3	1	+2
Bad character	7.4	72%	7.2	62%	17	5	+12
Weak character	7.8	89%	7.0	67%	4	2	+2
Hard to help	6.8	63%	7.0	62%	*n.a.*	*n.a.*	*n.a.*
Easy to identify	7.7	83%	6.9	59%	3	0	+3
Cannot lead useful lives	7.6	89%	6.8	69%	13	5	+8
Drink all the time	7.4	78%	6.5	71%	1	0	+1
Dangerous	6.4	53%	6.4	71%	27	5	+22
Simple cause	6.5	54%	6.2	62%	n.a.	n.a.	n.a.
Drink in predictable ways	5.9	51%	5.2	61%	n.a.	n.a.	n.a.

Note: Mean importance score based on an average of responses against a 9-point Likert: 1 (not at all important to include)—9 (very important to include). Consensus defined as 70% of responses falling within a three-point range within the Likert scale. Systematic literature review: count of attitudes that were either endorsed or not endorsed by myth category. n.a. relates to categories not identified in the literature review.

### Round 3 qualitative summary

In respondents’ qualitative Round 3 feedback, high importance scores were typically attributed to (1) accuracy of facts, (2) harmfulness of myths (e.g. ‘this allows people to ‘other’ people with AUD’) and (3) positive impact of facts (e.g. ‘this provides hope’).

Conversely, lower scores were attributed to (1) themes being better covered by other pairs (e.g. ‘weak character’ linking with other blame myths), (2) inaccurate factual information and (3) pairs lacking relevance to stigma (e.g. ‘drink in predictable ways’ and ‘simple cause’). Additional feedback was that the ‘can’t lead useful lives’ pair was not optimally phrased and that self-responsibility for change should be acknowledged when countering ‘to blame’ (‘important to acknowledge the individual still has the responsibility of overcoming their dependence’).

**Figure 3:**
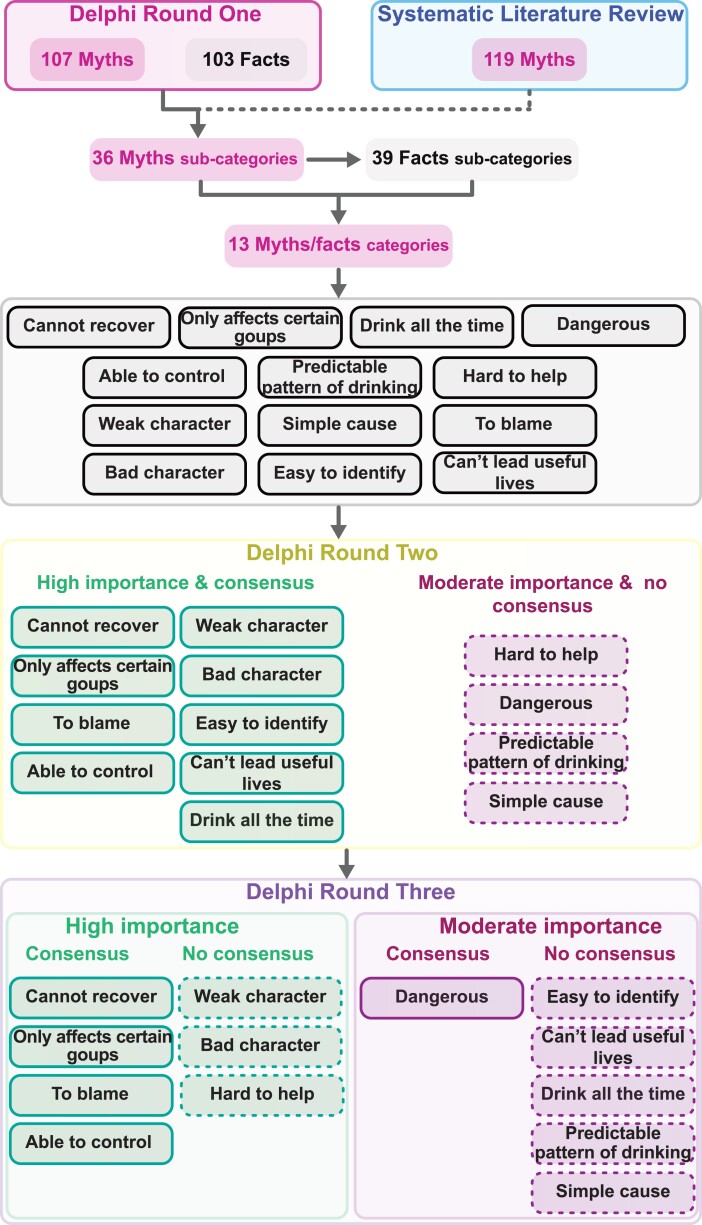
Flow-chart of myth-fact category generation and each Delphi Round.

Figure 3 presents an overall summary of the analysis for all rounds.

## Discussion

### Summary of findings and implications

To our knowledge, this is the first study to gather expert-led myths and facts to include in educational interventions for the UK public stigma of AUD. The use of a Delphi methodology allowed decision-making to be shared equally between professionals and experts by experience, which was important given that anti-stigma intervention development benefits from the participation of impacted communities.^5^

Out of the 13 myths and facts generated in the process, the four considered most important to include were ‘Cannot recover’, ‘Only affects certain groups’, ‘To blame’, ‘Able to control’. These reached consensus on their high importance with high and increasing consensus across rounds. Conversely, the lowest-scoring myth-fact pairs (‘Dangerous’, ‘Simple cause’, ‘Drink in predictable ways’) consistently scored low, with increasing consensus across rounds. With scores for the six other myth-fact pairs fluctuating more, these findings suggest a relatively stable consensus on the most and least impactful pairs.

Notably, experts perceived all pairs as at least moderately important to include, ranking three (‘Bad character’, ‘Weak character’ and ‘Hard to help’) as highly important in Round 3. A potential implication is that countering multiple myths may benefit anti-stigma interventions if these interventions are long enough to accommodate more myths. Specifically, some higher-ranked pairs (e.g. ‘To blame’) may be supplemented by lower-ranked ones (e.g. ‘Weak character’), as suggested by the experts’ consideration of their thematic similarities.

The study’s myth-fact pairs broadly related to four themes: (i) recovery (‘Cannot recover’, ‘Hard to help’; ‘Can’t lead useful lives’); (ii) responsibility (‘To blame’, ‘Able to control’, ‘Simple cause’, ‘Bad character’, ‘Weak character’); (iii) difference (‘Only affects certain groups’, ‘Easy to identify’, ‘Drink all the time’, ‘Drink in predictable ways’) and (iv) danger (‘Dangerous’). Recovery- and responsibility-focused pairs were typically viewed as more important. ‘Only affects certain groups’, a difference-focused pair scoring consistently highly, was an exception, even though it could be linked to responsibility as ‘othering’ people with a stigmatized condition can increase blame perceptions.^11^

Mixed alignment was found between myths identified in the systematic review as prevalent or strongly held by the public, and those considered important by experts. Echoing experts’ recommendations, responsibility-focused attitudes were found to be prevalent in the review. For example, at least half of the participants in a representative UK survey believed people with alcoholism had themselves to blame and could pull themselves together.^4^

However, difference-related stereotypes, (e.g.^4,12^) and beliefs that recovery is not possible^4,13^ were not widely endorsed in the literature, in contrast to the importance the experts attributed to them. Divergence also existed around danger-focused myths, which were frequently endorsed in the literature (e.g.^14^) but considered of lower importance by experts in the present study.

These discrepancies merit further investigation and underscore the need for an updated population study of UK public attitudes towards AUD to clarify current stereotypes. Nonetheless, previous research does suggest the highest-ranked myth-fact pairs carry the potential for stigma reduction. For example, countering the ‘only affects certain groups’ myth aligns with research finding that where the public perceives similarity between themselves and people with neuropsychiatric disorders, stigma reduces.^12^ Public stigma reduction following recovery-focused information supports the inclusion of ‘Cannot recover’ in an anti-stigma intervention.^15^ Additionally, challenging notions of ‘onset responsibility’ (i.e. responsibility for developing AUD) and ‘offset responsibility’ (i.e. responsibility for failure to recover) is recommended for stigma-reduction,^3^ indicating the importance of countering myths that people with AUD are ‘to blame for’, and ‘able to control’ their drinking.

Further, attribution theory provides theoretical support for targeting recovery- and responsibility-focused myths. With its pathways validated in AUD stigma, this holds that stigmatized conditions perceived as irreversible, controllable and caused by the individual lead to anger and diminished helping behaviour.

The present study illustrates challenges that should be considered when developing public AUD anti-stigma interventions. These include paradoxes in AUD stigma, such as that people with AUD are seen as ‘able to control’ their drinking, yet simultaneously lacking in self-control (i.e. ‘weak’). Interventions that over-emphasize some facts (e.g. impaired control) could inadvertently perpetuate harmful myths (e.g. recovery is not possible). Stereotypes that may ‘grow up from a kernel of truth’ pose additional challenges for message development. Highlighting this, panellists attributed lower rankings of danger-focused myths to stereotype accuracy. Balancing information that is both compassionate and realistic must, therefore, be a focus during AUD anti-stigma intervention development.

### Limitations

The study’s findings must be interpreted within the context of its limitations. First, myth-fact pairs were primarily assessed using quantitative scoring. This limits conclusions about the reasons for experts’ scores, which the quality of wording and factual information could have influenced. Second, despite the qualitative content analytic framework on which it was based, the selection of key themes and wording myth-fact pairs remained potentially influenced by the experimenter’s bias. Finally, while the Delphi method depends on the expertise of the panel, the sample size (16) and demographic characteristics (75% White British) of the present panel may hinder the generalization of the present findings to a broader population of experts. Therefore, the study’s myths and facts may not perfectly represent important experiences of stigma, which differ according to culture and context.

### Directions for future research

To build on this study’s findings, updated research is needed to clarify current UK public stereotypes about AUD that will allow better comparison between myths that are prevalent and those considered important by experts to include in public anti-stigma interventions. Further research could improve the wording of myth-fact pairs before intervention development. Data necessary to produce additional facts (e.g. rates of violence in AUD) may enhance the content quality since facts countering certain stereotypes (e.g. ‘violent’, ‘bad’ and ‘weak’) were difficult to retrieve in the present study.

Additional research into the optimal phrasing of the myth-fact pairs, which were drafted under time constraints in this study, could provide additional evidence to guide the development of future anti-stigma interventions. Further, quantitative evaluation of the relative impact of different myth-fact pairs would elucidate which messages are most effective for public stigma reduction.

Little is known about the most effective type of education intervention for AUD stigma-reduction. Future studies could, therefore, compare the effectiveness of different education interventions. These might include factual interventions,^8^ mental health literacy interventions, which provide education about the prevention and recognition of neuropsychiatric disorders, and awareness-raising interventions, which promote awareness of the prevalence and treatment of neuropsychiatric disorders.

Given the need to reduce the public stigma of all addictions, anti-stigma interventions focused on other addictive behaviours should be developed. This requires additional research, since different stereotypes characterize each addiction. Finally, education interventions found to be most effective should be implemented as part of an evaluated public campaign specifically targeting addiction-related stigma, given its relative persistence compared to that of other neuropsychiatric disorders following UK mental health campaigns to date.^4^

### Conclusion

This study aimed to expand the current evidence base for myth-fact interventions addressing public AUD stigma. The Delphi panel of professionals and experts by experience reached consensus on four myth-fact pairs that were more important to include in an anti-stigma intervention. These pairs related to responsibility- and recovery-based themes and were specifically that people with AUD ‘Cannot recover’, are ‘To blame’ for, and ‘Able to control’ their drinking, and that AUD ‘Only affects certain groups’. High importance was also ascribed to multiple other myth-fact pairs, suggesting inclusion of a range of stereotypes would be beneficial in future anti-stigma interventions. While existing research supports the potential of the most important myth-fact pairs to achieve stigma reduction, differences between experts’ views and the systematic review suggest a need for updated research into public attitudes towards AUD. The study’s findings can inform anti-stigma interventions for AUD and its approach applied to develop myth-fact interventions for other substance and behavioural addictions.

## Supplementary Material

fcaf035_Supplementary_Data

## Data Availability

Data are available at the Cambridge Repository: https://www.repository.cam.ac.uk/handle/1810/378475.
