# Supplementary material for: Myths and facts about alcohol use disorder: a Delphi consensus study
Source: Brain Commun. 2025 Jan 27;7(1):fcaf035. doi: 10.1093/braincomms/fcaf035 (PMC11806414; doi:10.1093/braincomms/fcaf035)
Supplement: fcaf035_Supplementary_Data [file fcaf035_supplementary_data.pdf]

SUPPLEMENTARY MATERIALS

SUPPLEMENTARY Figure 1

Round One Survey

Background Information

This study aims to identify a list of myths and facts about alcohol dependence to challenge the public stigma of alcohol dependence in the UK.

Round 1 Survey

Please answer Questions 1 and 2 by filling out the below myth-fact pairs.

Please fill out what you can from your knowledge and experience and do not worry if you need to leave boxes blank. Once you have finished, please click the red button at the bottom of the page to submit your responses.

	**Question 1** **1. In your experience, what are some common myths about alcohol dependence, and/or people with alcohol dependence?**<br><br>*Myths: false or overgeneralised beliefs*<br><br>Please write one myth per 'Myth' box below<br><br>**Myth 1**	**Question 2** **2. In your view, what corresponding fact (or facts) challenge each of the myths you have listed?**<br><br>*Challenge: demonstrate that each myth is false or overgeneralised*<br><br>Please write facts in the corresponding 'Fact' boxes alongside each myth If relevant, please mention references<br><br>**Fact(s) to challenge Myth 1**
1		

Etc.

SUPPLEMENTARY Figure 2

Round Two Survey

Introduction

Department of Psychology
City, University of London

Thank you for your ongoing contribution to the study, ‘Identifying 'myths and facts' to challenge the public stigma of alcohol dependence in the UK: a Delphi Study’.

Please read the following information before proceeding to the Round 2 Survey.

Recap on the purpose of the study

This Delphi study aims to identify ‘myths and facts’ to include in an intervention designed to reduce the public stigma of alcohol dependence in the UK. After the study completes, a myths and facts intervention will be created and tested on the general public to explore its effectiveness in improving public stigma towards people with alcohol dependence.

The planned intervention will be an educational video and will present the myths and facts about alcohol dependence using a narrator, animations and text.

What has happened since Round 1?

Following the Round 1 survey, your responses were anonymised and combined with those of other experts. This combined list of myth and fact statements was then analysed using qualitative analysis to identify common themes across all suggested myths and facts. Alongside this, a systematic literature review was conducted of studies of public attitudes towards alcohol dependence to identify further relevant themes.

Based on the themes appearing in the Round 1 survey responses and the literature review, a shortlist of myth-fact pairs was reached, which was used to create the Round 2 survey.

How do I complete the Round 2 Survey?

The aim of this round is to understand your initial view of the relative importance of the different myth-fact pairs for inclusion in an intervention aiming to reduce the public stigma of alcohol dependence.

In this survey, you will be presented with 13 myth-fact pairs and asked to rank them from 1-9 based on your view of their importance for inclusion in the intervention (1 = not at all important to include; 9 = very important to include).

Please click next to begin the Round 2 Survey.

Question 1
In your view, how important is the below myth-fact pair to include in an intervention aiming to reduce the public stigma of people with alcohol dependence?

NB. The intervention will be shown to the general public with a view to improving their attitudes, feelings and intended behaviour towards people with alcohol dependence.

Myth 1
People can't recover from alcohol dependence.
Fact(s) to challenge Myth 1
People can recover from alcohol dependence and go on to lead fulfilling lives.
Over half of people in treatment for alcohol use in alcohol services in England stop or reduce their drinking after 6 months of treatment.

Not at all important to include								*Very important to include*
1	*2*	*3*	*4*	*5*	*6*	*7*	*8*	*9*
☐	☐	☐	☐	☐	☐	☐	☐	☐

Etc.

SUPPLEMENTARY Figure 3

Round 3 Survey

Introduction

Department of Psychology
City, University of London

Thank you for your ongoing contribution to the study, ‘Identifying 'myths and facts' to challenge the public stigma of alcohol dependence in the UK: a Delphi Study’.

Please read the following information before proceeding to the Round 3 survey.

<u>Recap on the purpose of the study</u>

This Delphi study aims to identify ‘myths and facts’ to include in an intervention designed to reduce the public stigma of alcohol dependence in the UK. After the study completes, a myths and facts intervention will be created and tested on the general public to explore its effectiveness in improving public stigma towards people with alcohol dependence.

In this study, the intervention will be an educational video and will present the myths and facts about alcohol dependence using a narrator, animations and text.

<u>What has happened since Round 2?</u>

Following the Round 2 survey, panellists’ scores were weighted to ensure the views of each expert group on the panel are represented equally in the study’s findings. The weighted scores were then analysed to establish the average and distribution of scores across panellists. This analysis was used to create the Round 3 survey.

<u>How do I complete the Round 3 Survey?</u>

In this survey, you will again be asked to rank the 13 myth-fact pairs from 1-9 based on your view of their importance for inclusion in the intervention, this time considering summaries of the panel’s scores from Round 2.

You do not have to agree with other panellists and should continue to score the statements as you believe is most accurate based on your experience and expertise but are encouraged to consider the panel’s views from Round 2 before submitting your scores.

You will also have the opportunity to provide a reason for your score for each myth-fact pair – providing this additional feedback is optional. This feedback will be used to provide additional context to help explain the panel’s rankings in the study’s write up.

Please click next to begin the Round 3 Survey.

Question 1

In your view, how important is the below myth-fact pair to include in an intervention aiming to reduce the public stigma of people with alcohol dependence?

Please consider the panel's scores from Round 2 below before submitting your Round 3 score.
NB. The intervention will be shown to the general public with a view to improving their attitudes, feelings and intended behaviour towards people with alcohol dependence.

Myth 1

People can't recover from alcohol dependence.

Fact(s) to challenge Myth 1

People can recover from alcohol dependence and go on to lead fulfilling lives.

Over half of people in treatment for alcohol use in alcohol services in England stop or reduce their drinking after 6 months of treatment.

Round 2 Scores

Breakdown of scores
This table shows the percentage of panellists selecting each importance score for this myth-fact pair in Round 2.

Not at all important to include 1	2	3	4	5	6	7	8	*Very important to include* 9
0%	0%	0%	0%	7%	7%	0%	8%	78%

<u>*Average Round 2 importance score for this myth-fact pair*</u>

8.5

Please submit your Round 3 score for this myth-fact pair below:

Not at all important to include 1	2	3	4	5	6	7	8	*Very important to include* 9
☐	☐	☐	☐	☐	☐	☐	☐	☐

Please provide a reason for your score in the text box below *(optional)*:

Etc.

SUPPLEMENTARY Table 1

Key Findings from Systematic Literature Review

Author	Year	Geography	Stigma Type	Sample Size	Attitude recorded	Sub-category	Category	Stereotype endorsed?
Abdullah & Brown	2020	US	Personal	106	Measured fear/dangerousness, including assessment of dangerousness beliefs about an alcohol dependence vignette character e.g. 'How dangerous would you feel James is?, I think James poses a risk to his neighbors unless he is hospitalized	Risk to others	Dangerous	Not Endorsed (Quant)
Boysen et al.	2014	US	Personal	115	Participants rated alcohol use disorder according to how 'masculine' they thought the disorder was	Men/old men	Only affects certain groups	Endorsed (Quant)
					People with alcohol use disorder are dangerous; I would feel threatened by a person with alcohol use disorder	Risk to others	Dangerous	Endorsed (Quant)
Crisp et al.	2005	UK	Personal	1725	Danger to others	Risk to others	Dangerous	Endorsed (Quant)
					Unpredictable	Unpredictable	Dangerous	Endorsed (Quant)
					Feel different from us	Different	Only affects certain groups	Not Endorsed (Quant)
					Selves to blame	Caused by choice	To blame	Endorsed (Quant)
					Could pull self together	Could stop if wanted	Able to control	Endorsed (Quant)
					Not improved if treated	Recovery is impossible	Cannot recover	Not Endorsed (Quant)
					Never fully recover	Recovery is impossible	Cannot recover	Not Endorsed (Quant)
DePierre et al.	2013	US	Personal	570	Alcohol addiction is best seen as a form of wrongdoing	Bad	Bad Character	Not Endorsed (Quant)
					People become alcohol addicts because of moral weakness	Caused by character	To blame	Not Endorsed (Quant)
					People become alcohol addicts because they have weak, inadequate personalities	Caused by character	To blame	Not Endorsed (Quant)
Griffin et al.	2020	Tanzania	Perceived	340	Most people believe that a person who has had alcohol treatment is just as intelligent as the average person	Unintelligent	Can't lead useful lives	Endorsed (Quant)*
					Most people believe that a former alcoholic is just as trustworthy as the average person	Untrustworthy	Bad Character	Endorsed (Quant)*
					Most people feel that entering alcohol treatment is a sign of personal failure	Weak	Weak character	Not Endorsed (Quant)
					People who drink excessive alcohol are a loss to the community	Don't contribute	Can't lead useful lives	Endorsed (Qual)
					People who drink excessive alcohol are...considered bad people	Bad	Bad Character	Endorsed (Qual)
Horch & Hodgins	2008	Canada	Personal	249	How likely is it that Brandon (alcohol dependence vignette character) would do something violent to other people?	Risk to others	Dangerous	Not Endorsed (Quant)
					In your opinion, how likely is it that Brandon's (alcohol dependence vignette character's) situation might be because of his own bad character?	Caused by character	To blame	Endorsed (Quant)
Jahnke et al.	2015	Germany	Personal	854	Drinking large amounts of alcohol almost daily is something that one can choose.	Caused by choice	To blame	Endorsed (Quant)
					People who drink large amounts of alcohol almost daily have taken a deliberate decision to have these interests	Caused by choice	To blame	Not Endorsed (Quant)
					People have the choice whether they drink large amounts of alcohol almost daily or not	Caused by choice	To blame	Endorsed (Quant)
					A person who drinks large amounts of alcohol almost daily poses a danger to children	Risk to others	Dangerous	Endorsed (Quant)

					A person who drinks large amounts of alcohol almost daily poses a danger to adolescents	Risk to others	Dangerous	Endorsed (Quant)
					A person who drinks large amounts of alcohol almost daily poses a danger to adults	Risk to others	Dangerous	Endorsed (Quant)
Konkolÿ Thege et al.	2015	Canada	Personal	4000	In your opinion, how likely is it that an alcohol problem might be caused by a character problem or flaw?	Caused by character	To blame	Endorsed (Quant)
Koski-Jännes et al.	2012	Finland	Personal	1338	Participants were asked to indicate the degree to which the individual vs. circumstances beyond his or her control is responsible for causing alcohol dependence	Caused by choice	To blame	Endorsed (Quant)
					Participants were asked to indicate the chances of self-change in alcohol dependence	Could stop if wanted	Able to control	Endorsed (Quant)
Mackert et al.	2014	US	Personal	233	In your opinion, how likely is it that John's (alcohol dependence vignette character's) situation might be caused by his own bad character?	Caused by character	To blame	Endorsed (Quant)
					In your opinion, how likely is it that John (alcohol dependence vignette character) would do something violent toward other people?	Risk to others	Dangerous	Endorsed (Quant)
McCallum et al.	2016	Australia	Perceived	34	'I want people to know that I'm not just a homeless bum on the street who is an alcoholic. I mean I'm 32 and I'm just a standard young female who suffers from chronic alcoholism.'	Homeless/deprived	Only affects certain groups	Endorsed (Qual)
					About being an alcoholic: 'I want people to know...I am a sick person, not a bad person.'	Bad	Bad Character	Endorsed (Qual)
Meier et al.	2020	Tanzania	Perceived	104	Referring to the alcoholic in society: 'He will be isolated because he has been useless'	Don't contribute	Can't lead useful lives	Endorsed (Qual)
					About people who drink excessively: 'the community has seen him like a mad person.'	Chaotic	Can't lead useful lives	Endorsed (Qual)
Meurk et al.	2014	Australia	Personal	1263	Participants rated their agreement to 'bad character' being a cause of alcohol addiction	Caused by character	To blame	Endorsed (Quant)
Morgiève et al.	2019	France	Personal	2600	Do you think this type of problem [alcohol addiction] increases the risk of committing murder?	Risk to others	Dangerous	Endorsed (Quant)
					Do you think this type of problem [alcohol addiction] increases the risk of harming persons close?	Risk to others	Dangerous	Endorsed (Quant)
					Do you think this type of problem [alcohol addiction] increases the risk of being violent?	Risk to others	Dangerous	Endorsed (Quant)
					Do you think this type of problem [alcohol addiction] increases the risk of incurring debt?	Chaotic	Can't lead useful lives	Endorsed (Quant)
					Do you think this type of problem [alcohol addiction] increases the risk of committing suicide?	Risk to self	Dangerous	Endorsed (Quant)
Peluso & Blay	2008	Brazil	Personal	457	Participants were asked how far they agreed that 'weakness of character' was a cause of alcohol dependence	Caused by character	To blame	Endorsed (Quant)
					Participants were asked how far they agreed that 'lack of faith in God' was a cause of alcohol dependence	Caused by character	To blame	Endorsed (Quant)
					In your opinion, could a person like José (alcohol dependence vignette character) commit a violent act against other people?	Risk to others	Dangerous	Endorsed (Quant)
					If a person like José (alcohol dependence vignette character) was receiving appropriate treatment, do you believe that he could commit a violent act against other people?	Risk to others	Dangerous	Not Endorsed (Quant)
Perry et al.	2020	US	Personal	1169	Participants were asked how probable it is that the alcohol dependence vignette character would do something violent toward other people	Risk to others	Dangerous	Endorsed (Quant)
					Participants were asked how probable it is that the alcohol dependence vignette character would do something violent toward him or herself	Risk to self	Dangerous	Endorsed (Quant)

					Participants were asked how probable it is that the alcohol dependence vignette character can manage their money	Chaotic	Can't lead useful lives	Not Endorsed (Quant)*
					Participants were asked how probable it is that the alcohol dependence vignette character can make treatment decisions on their own	Chaotic	Can't lead useful lives	Not Endorsed (Quant)*
					Participants were asked how probable it is that the alcohol dependence vignette character's condition is caused by bad character	Caused by character	To blame	Endorsed (Quant)
Pescosolido et al.	2010	US	Personal	630	How likely is it that the person in the alcohol dependence vignette would 'do something violent toward other people'?	Risk to others	Dangerous	Endorsed (Quant)
					How likely is it that the person in the alcohol dependence vignette would 'do something violent toward him/herself'?	Risk to self	Dangerous	Endorsed (Quant)
					How likely is it that the situation [in the alcohol dependence vignette] might be caused by 'his or her own bad character'?	Caused by character	To blame	Endorsed (Quant)
Piras et al.	2016	Italy	Personal	404	Participants were asked to indicate to what extent they considered a person with alcohol addiction 'Clean' vs 'Dirty'	Unclean	Easy to identify	Endorsed (Quant)
					Participants were asked to indicate to what extent they considered a person with alcohol addiction 'Nice' vs 'Nasty'	Bad	Bad Character	Endorsed (Quant)
					Participants were asked to indicate to what extent they considered a person with alcohol addiction 'Industrious' vs 'Lazy'	Don't contribute	Can't lead useful lives	Endorsed (Quant)
					Participants were asked to indicate to what extent they considered a person with alcohol addiction 'Common' vs 'Strange'	Different	Only affects certain groups	Endorsed (Quant)
					Participants were asked to indicate to what extent they considered a person with alcohol addiction 'Pretty' vs 'Ugly'	Bad	Bad Character	Endorsed (Quant)
					Participants were asked to indicate to what extent they considered a person with alcohol addiction 'Ingenuous' vs 'Cunning'	Untrustworthy	Bad Character	Not Endorsed (Quant)
					Participants were asked to indicate to what extent they considered a person with alcohol addiction 'Pleasant' vs 'Unpleasant'	Bad	Bad Character	Endorsed (Quant)
					Participants were asked to indicate to what extent they considered a person with alcohol addiction 'Civilised' vs 'Uncivilised'	Bad	Bad Character	Endorsed (Quant)
					Participants were asked to indicate to what extent they considered a person with alcohol addiction 'Active' vs 'Passive'	Don't contribute	Can't lead useful lives	Endorsed (Quant)
					Participants were asked to indicate to what extent they considered a person with alcohol addiction 'Good' vs 'Bad'	Bad	Bad Character	Endorsed (Quant)
					Participants were asked to indicate to what extent they considered a person with alcohol addiction 'Simple' vs 'Complicated'	Chaotic	Can't lead useful lives	Endorsed (Quant)
					Participants were asked to indicate to what extent they considered a person with alcohol addiction 'Innocuous' vs 'Dangerous'	Risk to others	Dangerous	Endorsed (Quant)
					Participants were asked to indicate to what extent they considered a person with alcohol addiction 'Faithful' vs 'Unfaithful'	Untrustworthy	Bad Character	Endorsed (Quant)
					Participants were asked to indicate to what extent they considered a person with alcohol addiction 'Honest' vs 'Dishonest'	Untrustworthy	Bad Character	Endorsed (Quant)
					Participants were asked to indicate to what extent they considered a person with alcohol addiction 'Sincere' vs 'False'	Untrustworthy	Bad Character	Endorsed (Quant)
					Participants were asked to indicate to what extent they considered a person with alcohol addiction 'Calm' vs 'Agitated'	Unpredictable	Dangerous	Endorsed (Quant)

					Participants were asked to indicate to what extent they considered a person with alcohol addiction 'Right' vs 'Wrong'	Bad	Bad Character	Endorsed (Quant)
					Participants were asked to indicate to what extent they considered a person with alcohol addiction 'Clear' vs 'Dark'	Bad	Bad Character	Endorsed (Quant)
Schomerus et al.	2011	US	Perceived	26	'Unreliable'	Untrustworthy	Bad Character	Endorsed (Qual)
					'Emotionally unstable'	Unpredictable	Dangerous	Endorsed (Qual)
					'Violent'	Risk to others	Dangerous	Endorsed (Qual)
					'Living on other people's expenses'	Don't contribute	Can't lead useful lives	Endorsed (Qual)
					'Self-pitying'	Selfish	Bad Character	Endorsed (Qual)
					'Lazy'	Don't contribute	Can't lead useful lives	Endorsed (Qual)
					'Resolving conflicts only with alcohol'	Weak	Weak character	Endorsed (Qual)
					'Weak-willed'	Lack willpower	Weak character	Endorsed (Qual)
					'Unable to ever get away from alcohol'	Round the clock drinking	Drink all the time	Endorsed (Qual)
					'Unable to keep a regular job'	Chaotic	Can't lead useful lives	Endorsed (Qual)
					'To blame for their problems'	Caused by choice	To blame	Endorsed (Qual)
					'Not to be trusted'	Untrustworthy	Bad Character	Endorsed (Qual)
					'Disgusting'	Unclean	Easy to identify	Endorsed (Qual)
					'Dirty and Unkempt'	Unclean	Easy to identify	Endorsed (Qual)
					'Below average intelligence'	Unintelligent	Can't lead useful lives	Endorsed (Qual)
					'Unpredictable'	Unpredictable	Dangerous	Endorsed (Qual)
Schomerus et al.	2006	Germany	Personal	1012	How strongly do you think it depends on oneself as to whether one gets alcoholism or not?	Caused by choice	To blame	Endorsed (Quant)
					How effective is, in your view, the treatment available nowadays for alcoholism?	Recovery is impossible	Cannot recover	Not Endorsed (Quant)*
					How great is your own risk of getting alcoholism?	Different	Only affects certain groups	Endorsed (Quant)*
Schomerus et al.	2013	Germany	Personal	3642	In relation to an alcohol dependence vignette character: Basically we are all sometimes like this person. It’s just a question how pronounced this state is.	Different	Only affects certain groups	Not Endorsed (Quant)*
Schomerus et al.	2014a	Germany	Personal	1167	Alcoholism results from weakness of character	Caused by character	To blame	Not Endorsed (Quant)
					Alcoholics are themselves to blame for their bad fate	Caused by choice	To blame	Not Endorsed (Quant)
					With more discipline, alcoholics could control their drinking and drink normally	Could control if tried	Able to control	Not Endorsed (Quant)
Schomerus et al.	2014b	Germany	Perceived	967	Participants asked the extent to which they believed the general public would endorse 'Short-tempered' as an attribute of alcohol dependent patients	Unpredictable	Dangerous	Endorsed (Quant)
					Participants asked the extent to which they believed the general public would endorse 'Unpredictable' as an attribute of alcohol dependent patients	Unpredictable	Dangerous	Endorsed (Quant)
					Participants asked the extent to which they believed the general public would endorse 'Aggressive' as an attribute of alcohol dependent patients	Risk to others	Dangerous	Endorsed (Quant)
					Participants asked the extent to which they believed the general public would endorse 'Dependent on others' as an attribute of alcohol dependent patients	Don't contribute	Can't lead useful lives	Endorsed (Quant)
					Participants asked the extent to which they believed the general public would endorse 'Strange' as an attribute of alcohol dependent patients	Different	Only affects certain groups	Not Endorsed (Quant)

					Participants asked the extent to which they believed the general public would endorse 'Stupid' as an attribute of alcohol dependent patients	Unintelligent	Can't lead useful lives	Not Endorsed (Quant)
					Participants asked the extent to which they believed the general public would endorse 'Scary' as an attribute of alcohol dependent patients	Risk to others	Dangerous	Not Endorsed (Quant)
					Participants asked the extent to which they believed the general public would endorse 'Untruthful' as an attribute of alcohol dependent patients	Untrustworthy	Bad Character	Not Endorsed (Quant)
					Participants asked the extent to which they believed the general public would endorse 'Dangerous' as an attribute of alcohol dependent patients	Risk to others	Dangerous	Not Endorsed (Quant)
					Most people believe that a person who has been in a hospital for alcohol detoxification is just as intelligent as the average person	Unintelligent	Can't lead useful lives	Not Endorsed (Quant)*
					Most people believe that someone who has been treated for alcoholism is just as trustworthy as the average citizen	Untrustworthy	Bad Character	Not Endorsed (Quant)*
Sorsdahl et al.	2012	South Africa	Personal	868	Participants asked to indicate whether they thought the behaviour described in the alcohol use disorder vignette was typical of a weak character,	Weak	Weak character	Endorsed (Quant)
					How dangerous would you feel Jeremy (alcohol use disorder vignette character) is?	Risk to others	Dangerous	Endorsed (Quant)
					I would think that it was Jeremy's (alcohol use disorder vignette character's) own fault that he is in the present condition	Caused by choice	To blame	Endorsed (Quant)
Staton et al.	2018	Tanzania	Perceived	35	Most people believe that a person who has had alcohol treatment is just as intelligent as the average person	Unintelligent	Can't lead useful lives	Not Endorsed (Quant)*
					Most people believe that a former alcoholic is just as trustworthy as the average person	Untrustworthy	Bad Character	Not Endorsed (Quant)*
					Most people feel that entering alcohol treatment is a sign of personal failure	Weak	Weak character	Not Endorsed (Quant)
Subramaniam et al.	2017a	Singapore	Personal	3000	People with a problem like [alcohol abuse vignette character]'s could get better if they wanted to	Could stop if wanted	Able to control	Endorsed (Quant)
					A problem like [alcohol abuse vignette character]'s is a sign of personal weakness	Weak	Weak character	Endorsed (Quant)
					People with a problem like [alcohol abuse vignette character]'s are dangerous to others	Risk to others	Dangerous	Endorsed (Quant)
					People with a problem like [alcohol abuse vignette character]'s are unpredictable	Unpredictable	Dangerous	Endorsed (Quant)
Subramaniam et al.	2017b	Singapore	Personal	3006	In relation to an alcohol abuse vignette character: Basically we are all sometimes like this person. It’s just a question how pronounced this state is	Different	Only affects certain groups	Not Endorsed (Quant)*
To & Vega	2006	US	Personal	~1000	Alcoholism is caused, at least in part, by moral weakness	Caused by character	To blame	Endorsed (Quant)
Toornstra et al.	2020	Ukraine	Perceived	38	The people around me did not trust me anymore'	Untrustworthy	Bad Character	Endorsed (Qual)
Valentine & Walter	2015	UK	Perceived	6	A lot of people think well he caused his own death'	Caused by choice	To blame	Endorsed (Qual)

Systematic Review References

Abdullah, T., & Brown, T. L. (2020). Diagnostic labeling and mental illness stigma among Black Americans: An experimental vignette study. *Stigma and Health*, *5*(1), 11-21. https://doi.org/10.1037/sah0000162

Boysen, G., Ebersole, A., Casner, R., & Coston, N. (2014). Gendered mental disorders: masculine and feminine stereotypes about mental disorders and their relation to stigma. *J Soc Psychol*, *154*(6), 546-565. https://doi.org/10.1080/00224545.2014.953028

Crisp, A., Gelder, M., Goddard, E., & Meltzer, H. (2005). Stigmatization of people with mental illnesses: a follow-up study within the Changing Minds campaign of the Royal College of Psychiatrists. *World psychiatry*, *4*(2), 106-113.

DePierre, J. A., Puhl, R. M., & Luedicke, J. (2013). Public perceptions of food addiction: a comparison with alcohol and tobacco. *Journal of Substance Use*, *19*(1-2), 1-6. https://doi.org/10.3109/14659891.2012.696771

Griffin, S. M., Karia, F. P., Zimmerman, A., Minnig, M. C. C., Swahn, M., Makelarski, J., B, T. M., Vissoci, J. R. N., & C, A. S. (2020). A Mixed-Methods Study: Sex Differences in Experiences of Stigma Associated With Alcoholism and Alcohol Use Disorders Among Injury Patients in Tanzania. *Alcohol Clin Exp Res*, *44*(8), 1700-1707. https://doi.org/10.1111/acer.14402

Horch, J. D., & Hodgins, D. C. (2008). Public Stigma Of Disordered Gambling : Social Distance, Dangerousness, And Familiarity *Journal of social and clinical psychology*, *27*(5), 505-528. https://doi.org/10.1521/jscp.2008.27.5.505

Jahnke, S., Imhoff, R., & Hoyer, J. (2015). Stigmatization of people with pedophilia: two comparative surveys. *Arch Sex Behav*, *44*(1), 21-34. https://doi.org/10.1007/s10508-014-0312-4

Konkoly Thege, B., Colman, I., el-Guebaly, N., Hodgins, D. C., Patten, S. B., Schopflocher, D., Wolfe, J., & Wild, T. C. (2015). Social judgments of behavioral versus substance-related addictions: a population-based study. *Addict Behav*, *42*, 24-31. https://doi.org/10.1016/j.addbeh.2014.10.025

Koski-Jannes, A., Hirschovits-Gerz, T., & Pennonen, M. (2012). Population, professional, and client support for different models of managing addictive behaviors. *Subst Use Misuse*, *47*(3), 296-308. https://doi.org/10.3109/10826084.2011.629708

Mackert, M., Mabry, A., Hubbard, K., Grahovac, I., & Holleran Steiker, L. (2014). Perceptions of Substance Abuse on College Campuses: Proximity to the Problem, Stigma, and Health Promotion. *Journal of Social Work Practice in the Addictions*, *14*(3), 273-285. https://doi.org/10.1080/1533256x.2014.936247

McCallum, S. L., Mikocka-Walus, A. A., Gaughwin, M. D., Andrews, J. M., & Turnbull, D. A. (2016). 'I'm a sick person, not a bad person': patient experiences of treatments for alcohol use disorders. *Health Expect*, *19*(4), 828-841. https://doi.org/10.1111/hex.12379

Meier, B. J., El-Gabri, D., Friedman, K., Mvungi, M., Mmbaga, B. T., Nickenig Vissoci, J. R., & Staton, C. A. (2020). Perceptions of alcohol use among injury patients and their family members in Tanzanian society. *Alcohol*, *83*, 9-15. https://doi.org/10.1016/j.alcohol.2019.06.001

Meurk, C., Carter, A., Partridge, B., Lucke, J., & Hall, W. (2014). How is acceptance of the brain disease model of addiction related to Australians' attitudes towards addicted individuals and treatments for addiction? *BMC Psychiatry*, *14*, 373. https://doi.org/10.1186/s12888-014-0373-x

Morgiève, M., N'Diaye, K., Nguyen-Khac, A., Mallet, L., & Briffault, X. (2019). Crazy'App: A web survey on representations and attitudes toward mental disorders using video testimonies. *Encephale*, *45*(4), 290-296. https://doi.org/10.1016/j.encep.2018.10.004

Peluso, E. d. T. P., & Blay, S. L. (2008). Public perception of alcohol dependence. *Revista brasileira de psiquiatria*, *30*(1), 19-24. https://doi.org/10.1590/s1516-44462008000100004

Perry, B. L., Pescosolido, B. A., & Krendl, A. C. (2020). The unique nature of public stigma toward non-medical prescription opioid use and dependence: a national study. *Addiction*, *115*(12), 2317-2326. https://doi.org/10.1111/add.15069

Pescosolido, B. A., Martin, J. K., Long, J. S., Medina, T. R., Phelan, J. C., & Link, B. G. (2010). "A Disease Like Any Other"? A Decade of Change in Public Reactions to Schizophrenia, Depression, and Alcohol Dependence. *The American journal of psychiatry*, *167*(11), 1321-1330. https://doi.org/10.1176/appi.ajp.2010.09121743

Piras, A. P., Preti, A., Moro, M. F., Giua, A., Sini, G., Piras, M., Pintus, M., Pintus, E., Manca, A., Cannas, G., Cossu, G., Angermeyer, M. C., & Carta, M. G. (2016). Does calling alcoholism an illness make a difference? The public image of alcoholism in Italy. *Drug Alcohol Depend*, *166*, 39-44. https://doi.org/10.1016/j.drugalcdep.2016.06.015

Schomerus, G., Corrigan, P. W., Klauer, T., Kuwert, P., Freyberger, H. J., & Lucht, M. (2011). Self-stigma in alcohol dependence: consequences for drinking-refusal self-efficacy. *Drug Alcohol Depend*, *114*(1), 12-17. https://doi.org/10.1016/j.drugalcdep.2010.08.013

Schomerus, G., Matschinger, H., & Angermeyer, M. C. (2006). Preferences of the public regarding cutbacks in expenditure for patient care: are there indications of discrimination against those with mental disorders? *Soc Psychiatry Psychiatr Epidemiol*, *41*(5), 369-377. https://doi.org/10.1007/s00127-005-0029-8

Schomerus, G., Matschinger, H., & Angermeyer, M. C. (2013). Continuum beliefs and stigmatizing attitudes towards persons with schizophrenia, depression and alcohol dependence. *Psychiatry Res*, *209*(3), 665-669. https://doi.org/10.1016/j.psychres.2013.02.006

Schomerus, G., Matschinger, H., & Angermeyer, M. C. (2014a). Attitudes towards alcohol dependence and affected individuals: persistence of negative stereotypes and illness beliefs between 1990 and 2011. *Eur Addict Res*, *20*(6), 293-299. https://doi.org/10.1159/000362407

Schomerus, G., Matschinger, H., Lucht, M. J., & Angermeyer, M. C. (2014b). Changes in the perception of alcohol-related stigma in Germany over the last two decades. *Drug Alcohol Depend*, *143*, 225-231. https://doi.org/10.1016/j.drugalcdep.2014.07.033

Sorsdahl, K., Stein, D. J., & Myers, B. (2012). Negative attributions towards people with substance use disorders in South Africa: variation across substances and by gender. *BMC Psychiatry*, *12*(1), 101-101. https://doi.org/10.1186/1471-244X-12-101

Staton, C. A., Vissoci, J. R. N., Wojcik, R., Hirshon, J. M., Mvungi, M., Mmbaga, B. T., & Swahn, M. (2018). Perceived barriers by health care providers for screening and management of excessive alcohol use in an emergency department of a low-income country. *Alcohol*, *71*, 65-73. https://doi.org/10.1016/j.alcohol.2018.01.003

Subramaniam, M., Abdin, E., Picco, L., Pang, S., Shafie, S., Vaingankar, J. A., Kwok, K. W., Verma, K., & Chong, S. A. (2017a). Stigma towards people with mental disorders and its components - a perspective from multi-ethnic Singapore. *Epidemiol Psychiatr Sci*, *26*(4), 371-382. https://doi.org/10.1017/S2045796016000159

Subramaniam, M., Abdin, E., Picco, L., Shahwan, S., Jeyagurunathan, A., Vaingankar, J. A., & Chong, S. A. (2017b). Continuum beliefs and stigmatising beliefs about mental illness: results from an Asian community survey. *BMJ Open*, *7*(4), e014993. https://doi.org/10.1136/bmjopen-2016-014993

To, S. E., & Vega, C. P. (2006). Alcoholism and pathways to recovery: new survey results on views and treatment options. *Medscape general medicine*, *8*(1), 2.

Toornstra, A., Massar, K., Hurks, P. P. M., Timmermans, M., Kok, G., & Curfs, L. M. G. (2020). Perceptions of Alcohol and Alcohol Use among Community Members and Young Adults in Ukraine. *Subst Use Misuse*, *55*(8), 1269-1279. https://doi.org/10.1080/10826084.2020.1735436

Valentine, C., & Walter, T. (2015). Creative Responses to a Drug- or Alcohol-Related Death. *Illness, Crisis & Loss*, *23*(4), 310-322. https://doi.org/10.1177/1054137315590733

SUPPLEMENTARY Table 2

Key Findings from Delphi Round One and Systematic Literature Review

Myths (Round One and Systematic literature review)		Facts (Round One only)
**Category**	**Subcategories**	**Subcategories**
**Cannot recover**	Recovery is impossible	Recovery is possible<br>Sustained recovery<br>Recovery with support
	Can't lead happy lives*	More fulfilled lives
	Relapse is inevitable*	Relapse is not inevitable
	Physical damage is permanent*	Physical damage is reparable
**Only affects certain groups**	Homeless/deprived	Diverse socioeconomic groups
	Men/old men	Diverse genders and ages
	Different	Continuum of severity
**Drink all the time**	Daily drinking*	Dependence without daily drinking
	Round the clock drinking	Drink at varying times<br>Functioning
	Morning drinking*	Dependence without morning drinking
**Able to control**	Could stop if wanted	Require support to stop<br>Limited control
	Could control if tried	Physical dependence
**Drink in predictable ways***	Only drink spirits*	Type of drink varies
	Only drink in certain contexts*	Drinking context varies
	Drink large amounts*	Volume of drink varies
**Hard to help***	Limited options for recovery*	Various options for recovery
	Cannot support while drinking*	Can support while drinking
**Weak character**	Weak	Evidence of strengths
	Lack willpower	Demonstrate willpower<br>Other contributing factors
**Simple cause***	Alcohol abuse*	Other causal factors
	Trauma*	Trauma not always present
	Genetics*	Not always inherited
	Mental illness*	Mental illness not always present
**To blame**	Caused by choice	Various contributing factors
	Caused by character	Various contributing factors
**Bad character**	Selfish	Care about others
	Bad	Kind<br>Unfounded

	Untrustworthy	Honest
**Easy to identify**	Appear drunk	Tolerance varies<br>Not always visible
	Unclean+	
**Can't lead useful lives**	Chaotic	Can function
	Don't contribute	Contribute in recovery
	Unintelligent+	
**Dangerous**	Risk to others	Victims of violence
	Unpredictable+	
	Risk to self+	

*Note: For myth categories and subcategories: No marker = appeared in both Round One and the systematic literature review; *appeared only in Round One; +appeared only in the systematic literature review.*

Fact subcategories were sourced from Round One only
